# Sexual dysfunction among pregnant Tunisian women

**DOI:** 10.1192/j.eurpsy.2023.1337

**Published:** 2023-07-19

**Authors:** A. Zouari, F. Guermazi, B. Amamou, R. Masmoudi, I. Feki, I. Baati, J. Masmoudi, L. Gaha

**Affiliations:** 1 Psychiatry A, Hedi Chaker Hospital, sfax; 2 Psychiatry, EPS Fattouma Bourguiba, monastir, Tunisia

## Abstract

**Introduction:**

Sexual life is affected by physiological, psychological and social changes during pregnancy. Therefore, pregnancy is considered as a stressor affecting sexual lives of women and as a period when sexual dysfunctions can easly appear.

**Objectives:**

The aim of our study was to explore the prevalence of sexual dysfunctions among pregnant womens.

**Methods:**

we conducted a cross-sectional and descriptive study among Tunisian pregnant women. The questionnaire used was performed with Google Forms and posted on social media. It contained questions concerning personal and sociodemographic aspects and questions concerning obstetrical data such as parity, gestational age and complications during the current pregnancy. We used the Female Sexual Function Index to examine Sexual dysfunction. Total scores of 26.55 or less characterize deficiency of female sexual function.

**Results:**

Fifteen women (44.1%) were primiparous and 19 (55.9%) were multiparous with 29% being in the first trimester, 27% in the second, and 44% in the third. Half had at least one child. Most of participants reported better satisfaction with their sexuality before pregnancy than during pregnancy (76.5% vs. 26.5%). This difference in satisfaction was statistically significant (p=0.004). A sexual dysfunction was found in 70.6% of cases and we did not identify any correlations between the presence of sexual dysfunction and sociodemographic or clinical data of the current pregnancy.

**Conclusions:**

The prevalence of sexual dysfunction among Tunisian pregnant women was high and not related tosocio-demographic characteristics.

**Disclosure of Interest:**

None Declared

